# Porosity Formation Mechanisms and Their Effects on the Mechanical Properties of High-Density Polyethylene Extrusion Welds

**DOI:** 10.3390/polym18192360

**Published:** 2026-09-28

**Authors:** Suseong Woo, Jisun Kim

**Affiliations:** Korea Institute of Industrial Technology (KITECH), Cheomdan-gwagiro 208 beon-gil 6, Buk-gu, Gwangju 61012, Republic of Korea; mercury@kitech.re.kr

**Keywords:** high-density polyethylene (HDPE), extrusion welding, porosity formation, mechanical properties

## Abstract

High-density polyethylene (HDPE) has attracted attention as an alternative material for marine structures because of its chemical resistance, processability, and recyclability. However, internal pores formed during extrusion welding can reduce weld quality and mechanical performance. This study investigated pore formation mechanisms in HDPE extrusion welds by considering material throughput, filler wire storage condition, and artificial defect volume fraction. Porosity increased from 0.06% to 0.37% as material throughput increased from 0.96 to 2.00 kg/h and slightly decreased to 0.33% at 2.60 kg/h. Under these process conditions, tensile strength changed within approximately 1.49%, whereas elongation decreased more clearly with increasing porosity. In contrast, residual surface moisture on the filler wire caused severe pore formation and reduced tensile strength by approximately 55.53%. Artificial defect tests also confirmed that increasing internal defect volume directly reduced tensile strength. Overall, controlling material throughput and removing surface moisture are essential for improving HDPE extrusion weld quality.

## 1. Introduction

Recently, growing environmental concerns and the need for sustainable marine structures have increased interest in alternative materials for small vessels and other marine applications. Fiber-reinforced plastic (FRP) has been widely used in small boat construction because of its light weight and corrosion resistance [1]. However, FRP has limitations in terms of recyclability and end-of-life disposal [2]. In this regard, high-density polyethylene (HDPE) has attracted attention as a potential alternative material because of its chemical resistance, impact resistance, processability, and recyclability [3].

HDPE structures are generally fabricated by joining plate or profile components, and welding is therefore an important process for securing structural integrity. Among various thermoplastic welding methods, extrusion welding is suitable for joining relatively thick HDPE components because molten filler material can be continuously supplied into the weld zone while heat and pressure are applied [4]. For this reason, extrusion welding can be applied to large HDPE structures, including tanks, pipes, and marine components. However, the quality of extrusion welds is strongly affected by process parameters such as material throughput, melt temperature, preheating temperature, welding speed, and welding pressure [5].

One of the representative defects formed during thermoplastic welding is internal porosity [6]. Pores generated in the weld zone can reduce the effective load-bearing area and act as local stress concentration sites, which may deteriorate the mechanical performance of the welded joint [7]. In extrusion welding, pore formation can be affected by gas remaining in the molten material, insufficient degassing during plasticization, initial defects in the filler material, and moisture present on the surface of the filler wire [8]. In particular, because compact extrusion welding machines generally have a relatively short barrel length, the residence time for melting, mixing, and gas release may be limited [9]. Therefore, material throughput and the storage condition of the filler wire are expected to influence pore formation in HDPE extrusion welds.

Lee et al. investigated the effect of preheating parameters, including preheating temperature, hot-air movement speed, and nozzle distance, on the extrusion welding performance of HDPE materials. They reported that the preheating condition affects the heat distribution in the groove region and is an important factor for achieving reliable HDPE extrusion welds [10]. Rowe and Ali evaluated the effects of welding parameters on the properties of HDPE geomembrane extrusion welds. They reported that weld quality factors, such as welding temperature, bead geometry irregularity, and surface grinding condition, can statistically affect the stress crack resistance and variability of extrusion weld performance [11]. Balkan et al. investigated the effects of welding procedures on the mechanical and morphological properties of hot-gas butt-welded polyethylene (PE), polypropylene (PP), and polyvinyl chloride (PVC) sheets. They reported that the welding procedure and the use of a welding shoe significantly affected weld strength, and that PE and PP showed better weldability than PVC [12]. Zhang et al. studied the effects of welding parameters in dual-wedge welding on the physical, mechanical, and chemical properties of HDPE geomembrane seams. They reported that welding heat input, welding speed, and nip pressure influenced seam quality, and that the fusion area thickness and air-channel width could be used as indicators of welding quality [13]. Wang et al. investigated the tensile strength, ductility, crystallinity, and hardness of thick-walled HDPE pipe joints fabricated using different butt fusion welding processes. They reported that the mechanical properties of the welded joints varied depending on the welding process and weld location, and that the middle region of the joint showed lower tensile strength and fracture energy than the inner and outer regions [14]. Abdulrehman et al. investigated the mechanical properties of HDPE plates joined by friction stir welding under different tool rotational speeds. They reported that the welding parameters significantly affected the mechanical properties of the welded joints, indicating that process control is important for improving the weld strength of HDPE [15]. Mehdikhani et al. investigated orbital friction stir welding of HDPE pipes using a heating-assisted welding system and evaluated the mechanical and microstructural characteristics of the welded joints. They reported that sound and fully consolidated HDPE pipe joints could be obtained under appropriate welding conditions, demonstrating the applicability of friction stir welding to HDPE pipe joining [16].

Previous studies have examined how welding parameters and procedures influence the quality and mechanical properties of thermoplastic joints. However, the relationship between pore formation and tensile behavior in HDPE extrusion welds requires further clarification, particularly with respect to material throughput, residual surface moisture on the filler wire, and defect volume fraction. This study was guided by three hypotheses: changes in material throughput would alter pore formation by affecting barrel residence time and gas release; residual surface moisture on the filler wire would promote pore formation and impair joint tensile properties; and increasing the volume fraction of artificial defects fabricated using a consistent geometric design would reduce tensile strength.

To examine these hypotheses, this study combined radiographic testing (RT)-based characterization of pores with tensile testing of HDPE extrusion welds produced under different material throughput and filler wire storage conditions. Welding speed was adjusted with throughput to maintain comparable bead geometry. Complementary tensile tests were performed on HDPE base material containing artificial defects with controlled volumes. These defects provided a means of evaluating the relationship between defect volume fraction and tensile strength within a prescribed defect geometry, without implying mechanical equivalence to actual weld pores. The contribution of this study lies in bringing together observations of process- and moisture-related pore formation with controlled artificial defect testing to examine how pore characteristics relate to tensile behavior. This approach provides a basis for interpreting pore-related mechanical responses and improving quality control in HDPE extrusion welding.

## 2. Materials and Methods

### 2.1. HDPE Extrusion Welding System

HDPE extrusion welding is generally performed as a manual process and is therefore highly dependent on the operator. As a result, variations in weld quality can occur even under identical conditions, which limits the reproducibility and reliability of the experiments. To address this issue, an automated extrusion welding system based on a six-axis manipulator robot (Yaskawa Inc., Kitakyushu, Japan) was developed in this study, as shown in Figure 1A. By maintaining constant welding conditions through the automated system, higher reproducibility than manual welding was achieved. Accordingly, reliable experimental results could be obtained with a relatively small number of test repetitions.

HDPE extrusion welding using an extruder (Extruder D4, Sinwoo Inc., Seoul, Republic of Korea) is a welding process in which a filler wire is fed into the barrel, transported, cut, mixed, and melted by screw rotation, and the plasticized material is continuously extruded into the weld zone to form a joint. Figure 1B shows the HDPE extrusion welding process. The filler wire is melted by friction generated during screw rotation and by heaters installed inside the barrel, and the molten material is discharged through the nozzle into the weld zone. In addition, the welding shoe shapes the extruded filler material while transferring the extrusion pressure to the base material, thereby providing the joining pressure. The preheater was positioned ahead of the welding direction to preheat the surface of the base material and facilitate interfacial bonding. During the process, pressure and temperature were monitored using sensors, and the process parameters included material throughput, melt temperature, preheating temperature, welding speed, and welding pressure.

### 2.2. Determination of Welding Speed Based on Weld Zone Geometry and Material Throughput

In HDPE extrusion welding, the material throughput is determined by the rotational speed of the screw inside the extruder, and the material throughput and welding speed are closely related. Therefore, to form a weld zone with a constant cross-sectional area, the welding speed suitable for the material throughput should be determined. The welding speed was calculated using Equation (1).

(1) $$V_{w}=\frac{T}{10\rho A}$$

In Equation (1), V_w_ represents the welding speed, T represents the material throughput, ρ represents the density of the material, and A represents the cross-sectional area of the weld zone. The units of V_w_, T, ρ, and A are cm/min, g/min, g/mm^3^, and mm^2^, respectively.

The cross-sectional area of the weld zone was defined as the sum of the groove area and the bead area, as expressed in Equation (2).

A = A_1_ + A_2_ (2)

In Equation (2), A represents the cross-sectional area of the weld zone, A_1_ represents the groove area, and A_2_ represents the bead area.

The groove area was calculated considering the root gap and groove geometry, as expressed in Equation (3).

(3) $$A_{1}=g\cdot t+{\left(t-r\right)}^{2}\tan(\frac{\theta }{2})$$

In Equation (3), g represents the root gap, t represents the base material thickness, r represents the root face, and θ represents the groove angle. The units of g, t, and r are mm, and θ is expressed in degrees.

The bead area was calculated by simplifying the bead geometry as a triangle, as expressed in Equation (4).

(4) A2=12 bh

In Equation (4), b represents the bead width, and h represents the bead height. The units of b and h are mm.

### 2.3. Analysis Method for Pore Formation Mechanism

#### 2.3.1. Pore Analysis Method According to Process Conditions

In this study, material throughput was selected as the main process parameter to investigate pore formation in HDPE extrusion welding. Material throughput was controlled by adjusting the screw rotational speed using the speed control dial installed on the welding machine. In preliminary experiments, throughput was determined by measuring the mass of material extruded over 60 s under each condition. Each measurement was repeated three times, and the average value was used. The resulting material throughputs were 0.96, 2.0, and 2.6 kg/h. Welding speed was adjusted according to material throughput to maintain comparable bead geometry and weld volume for porosity evaluation. Although changes in welding speed can affect the thermal conditions of the base material and consequently the mechanical properties of the weld, our previous study [10] showed that the base material reached a temperature sufficient for melting at all investigated welding speeds, despite temperature differences of up to approximately 25%. These findings provided the basis for selecting the welding speeds used in this study. Welding was performed twice under each condition, and the detailed process conditions are presented in Table 1.

After welding, radiographic testing (RT) was performed on the welded specimens to observe the distribution of internal pores. The diameter of each pore was measured from the RT images, and its volume was estimated assuming a spherical shape. The estimated pore volumes were summed and divided by the total weld zone volume to calculate the porosity of each specimen. Subsequently, tensile tests were conducted to evaluate the mechanical properties of the weld zone in relation to porosity. The tensile tests were performed in accordance with EN 12814-2 [17], and a total of 10 tensile specimens were obtained from each welded specimen to capture the effects of pores as fully as possible.

#### 2.3.2. Pore Analysis Method According to Environmental Conditions

In this study, moisture absorption was selected as the main variable to analyze the pore formation characteristics of HDPE extrusion welds under different environmental conditions. To investigate the effect of moisture absorption, the HDPE filler wire was classified into three storage conditions, and the schematic diagram of the experiment is shown in Figure 2.

Case 1 represents the condition in which the filler wire was stored under general ambient conditions at room temperature. Case 2 represents the condition in which the filler wire was immersed in a water bath for a certain period and then used after removing surface moisture with a cloth. Case 3 represents the condition in which the filler wire was used without removing the surface moisture after storage in the water bath. The welding conditions used in the experiment are presented in Table 2.

Welding was performed twice under each condition. For the fabricated specimens, weld cross-sectional analysis and three tensile tests were conducted to evaluate the effects of moisture absorption in the filler wire and residual surface moisture on internal pore formation and mechanical properties of the welds.

#### 2.3.3. Evaluation of Porosity Effects Using Artificial Defects

Artificial defect specimens were fabricated to quantitatively evaluate the effect of internal pore volume on tensile strength. Although pores generated in actual extrusion welds generally have irregular shapes, the pores were simplified as spherical defects in this study to quantitatively control the defect volume. However, precisely forming spherical defects inside tensile specimens is limited in terms of machining process and dimensional control. Therefore, in this study, artificial defects were designed by converting spherical pores into cylindrical defects with equivalent volumes, as shown in Figure 3.

V_s_ = V_a_ (5)

(6) $$\frac{4}{3}\pi r^{3}=\pi r^{2}H+\frac{1}{3}\pi r^{2}\left(\frac{r}{\sqrt{\left(3\right)}}\right)$$

(7) H = r 43−133)

Equation (5) represents the volume equivalence between the spherical pore and the artificial defect. The artificial defects were formed at the center of the gauge length of the tensile specimens using CNC machining. Since a conical region was also formed at the bottom of the defect due to the tool tip geometry, the total volume of the artificial defect was calculated as the sum of the cylindrical volume and the conical volume. The tool tip angle was set to 120°, and the height of the conical region was assumed to be determined by the defect radius. Based on this geometry, Equation (6) was used to equate the volume of the spherical pore to the total volume of the artificial defect. Subsequently, the cylindrical defect depth, H, was derived as shown in Equation (7). Here, V_s_ is the volume of the spherical pore, V_a_ is the volume of the artificial defect, r is the defect radius, and H is the depth of the cylindrical region. Using these equations, artificial defects having volumes equivalent to spherical pores with diameters of 2, 4, and 6 mm were designed.

The fabricated artificial defect specimens were observed using an optical microscope to examine the surface morphology and defect diameter. As shown in Figure 4, the defect-free specimen and the artificial defect specimens with equivalent spherical pore diameters of 2, 4, and 6 mm were compared. The machined defects were formed at the intended position, corresponding to the center of the gauge length. In addition, the observation results confirmed that relatively uniform circular defect shapes were achieved under each condition. These results indicate that the designed volume-equivalent artificial defects were properly machined in the specimens. Subsequently, tensile tests were conducted to evaluate the variation in mechanical properties according to the defect volume fraction.

### 2.4. Materials

In this study, HDPE was used for both the base material and the filler wire. HDPE is a thermoplastic resin with high chemical resistance, excellent mechanical properties, and good recyclability, and is widely used in industrial applications such as piping systems, storage tanks, and marine structures. The marine-grade HDPE base plate was supplied by Eco Marine, and the HDPE filler wire was supplied by Röchling. Both materials were manufactured through extrusion processes. The base plate measured 495 mm × 115 mm × 12 mm, and the filler wire had a diameter of 4 mm. The melt index and thermal properties of each material are presented in Table 3.

### 2.5. Tensile Strength Evaluation Method for HDPE Welds

The strength of the HDPE extrusion welds was evaluated by tensile testing. To prepare specimens for mechanical property evaluation, tensile specimens were extracted from the central region of the welded plate after excluding 50 mm from both the start and end regions of the weld. This was done because the supply of molten material and the pressing condition can become unstable at the beginning and end of welding, which may result in variations in weld quality. Therefore, the central region, where a relatively stable welding condition was obtained, was selected for evaluation.

The extracted specimens were machined to remove the weld bead and prepare the surface, and tensile specimens with a final thickness of 10 mm were fabricated. A schematic diagram of the specimen extraction and preparation process is shown in Figure 5. The same specimen geometry was applied under all conditions, and the tensile tests were performed in accordance with EN 12814-2 using an Instron 34SC-5 testing machine (Instron, Norwood, MA, USA). The test speed was set to 50 mm/min.

## 3. Results and Discussion

### 3.1. Porosity Results According to Process Conditions

RT analysis revealed pores of various sizes within the welds, as shown in Figure 6. The pore diameters measured from the RT images ranged from approximately 1 to 5 mm. The volume of each pore was estimated from its measured diameter assuming a spherical shape. These volumes were summed and divided by the total weld volume to estimate the porosity of each specimen. The total weld volume was approximately 82,190 mm^3^, calculated from the average cross-sectional area of the weld bead and the welding length of 495 mm. The average estimated porosities were 0.06%, 0.37%, and 0.33% for Cases 1, 2, and 3, respectively. As shown in Figure 7, porosity did not increase monotonically with material throughput; it increased markedly from Case 1 to Case 2 and then decreased slightly in Case 3.

The higher estimated porosities in Cases 2 and 3 compared with Case 1 may be associated with insufficient gas release during melting and plasticization of the filler wire inside the barrel. HDPE extrusion welding machines have relatively short barrels compared with conventional injection molding machines or large-scale extrusion systems [18], which may limit the time available for gas release. Increased material throughput may further shorten the residence time of the molten material, allowing gases to enter the weld zone before being sufficiently released and remain as internal pores. This mechanism provides a possible explanation for the higher porosities observed at the higher throughputs, but does not by itself explain the slight decrease from Case 2 to Case 3.

If micropores are present in the filler wire, they may also remain incompletely eliminated during extrusion and contribute to pore formation in the weld zone. Under higher-throughput conditions, a shorter barrel residence time may limit the removal of both gases generated during plasticization and pre-existing pores in the filler wire. Thus, gas generation during processing and initial defects in the filler wire are possible contributors to the observed porosity.

For weld quality assessment, DVS 2202-1 [19] provides thickness-dependent acceptance criteria for pores and cavities in V-welds, double-V welds, and fillet welds. For acceptance level I, small isolated pores are permitted when Δs ≤ 0.1 s. For acceptance levels II and III, pores and rows of pores are permitted when Δs ≤ 0.15 s and Δs ≤ 0.2 s, respectively, where s is the material thickness and Δs is the defect dimension indicated in the standard. These criteria concern defect size relative to material thickness and are distinct from the volumetric porosity estimated in this study.

Pore formation may also depend on the extrusion welding machine configuration, barrel length, screw performance, and filler wire quality. Within the conditions investigated, the higher-throughput cases exhibited higher estimated porosities than Case 1, although the trend was not monotonic. Because welding speed was adjusted together with throughput, the individual effects of these parameters cannot be fully separated. Nevertheless, the findings support considering material throughput and the corresponding welding speed together when selecting process conditions to control pore formation. A longer barrel residence time at lower throughput may facilitate gas release and contribute to the lower porosity observed in Case 1.

The tensile test results are shown in Figure 8. The difference between the highest and lowest mean tensile strengths across the investigated conditions was approximately 1.49%. In particular, when comparing the material throughput conditions of 0.96 and 2.0 kg/h, the mean tensile strength increased slightly despite an approximately 492% increase in estimated porosity. Thus, the substantial relative change in estimated porosity was accompanied by only a small variation in the measured mean ultimate tensile strength within the experimental range.

In contrast, the elongation clearly decreased with increasing porosity, as shown in Figure 9. In particular, the 0.96 kg/h condition exhibited a larger variation in elongation than the other conditions. This is considered to result not simply from experimental error, but from the combined effects of the high ductility of HDPE and differences in weld quality. In other words, specimens with relatively few internal defects and sound bonding exhibited high elongation due to the pronounced ductile behavior inherent to HDPE. However, under conditions with increased porosity, the pores acted as stress concentration sites, reducing the deformation capacity of the weld zone [20]. Therefore, the increase in porosity is considered to have a more sensitive effect on elongation, that is, the ductility and deformation capacity before fracture of the weld zone, rather than on tensile strength [21].

### 3.2. Welding Results According to Environmental Conditions

The evaluation results of the welds according to the storage environment of the filler wire showed that the filler wire stored at room temperature (Case 1) and the filler wire immersed in water and then dried by removing surface moisture (Case 2) exhibited similar behavior in terms of weld surface and cross-sectional observations. In contrast, distinct pore formation was observed in the case of the filler wire used without removing surface moisture (Case 3). Figure 10 shows the surface morphology of the weld beads according to the storage conditions. Case 1 and Case 2 exhibited relatively uniform and stable surface morphologies, whereas Case 3 showed an uneven and rough surface due to internal pore formation.

The cross-sectional evaluation of the HDPE weld beads was performed according to the criteria specified in DVS 2202-1. This standard classifies defects in thermoplastic welds based on visual inspection and provides evaluation criteria, including requirements for bead shape, voids, undercuts, and notches. The bead height should satisfy the range of 0.1 s ≤ Height ≤ 0.3 s, where s is the base material thickness. For the 12 mm thick base material used in this study, the required bead height range is 1.2 mm ≤ Height ≤ 3.6 mm. Although no explicit numerical criterion is provided for the weld bead width, the bead should have a smooth profile and be free from notches. The experimental results showed that Case 1 and Case 2 exhibited similar tendencies in bead shape and width and satisfied the requirements of DVS 2202-1. In contrast, Case 3 showed a tendency for increased bead height along with the formation of numerous pores, indicating that excessive bead formation could exceed the specified range. The cross-sectional observation results of the welds are shown in Figure 11 and Table 4.

These changes in pore formation and bead geometry are considered to be caused by moisture present on the surface of the filler wire. When surface moisture was not removed, as in Case 3, the moisture evaporated under the high-temperature conditions during welding, locally expanding the molten material and simultaneously forming internal pores in the weld. In the HDPE extrusion welding process, the barrel temperature is maintained above approximately 240 °C [22]. Although some moisture evaporates during the process, excessive moisture may remain within the plasticized molten material. Subsequently, during extrusion and welding, the remaining moisture evaporates and appears as internal pores in the weld.

Meanwhile, HDPE is a nonpolar polymer composed only of carbon and hydrogen and has a very low moisture absorption rate, generally below 0.01% [23]. Therefore, even after water immersion, if the surface moisture is sufficiently removed before welding, weld quality similar to that obtained using filler wire stored at room temperature can be achieved. These results suggest that the moisture effect considered in this study is governed more by moisture present on the surface of the filler wire than by moisture absorption into the material.

Therefore, in the HDPE extrusion welding process, it is important to store the filler wire in an environment where humidity can be controlled. In particular, when the filler wire is exposed to moisture, weld quality can be improved by drying it using a dryer or oven, or by removing surface moisture with a cloth before welding.

The tensile strength evaluation results of the welds according to environmental conditions showed that internal pore defects were observed in some welds after specimen machining, and representative defect morphologies are shown in Figure 12. The tensile test results, shown in Figure 13, showed similar tensile strength levels for the condition using filler wire stored at room temperature and the condition using filler wire stored in water followed by surface moisture removal. In contrast, the condition using filler wire stored in water without removing surface moisture showed an approximately 55.53% decrease in tensile strength.

These results indicate that, in HDPE extrusion welding, the residual moisture present on the surface of the filler wire during welding has a more direct effect on weld strength than whether the filler wire had been exposed to moisture. The filler wire with removed surface moisture exhibited tensile strength similar to that of the filler wire stored under ambient conditions, despite its water immersion history. Therefore, the strength reduction is considered to be caused mainly not by water storage itself, but by moisture remaining on the surface of the filler wire during welding. This residual moisture evaporates due to heat during the welding process, forms pores inside the weld, and these pores reduce tensile strength by decreasing the effective cross-sectional area and acting as stress concentration sites.

In the previous evaluation of process conditions, the change in tensile strength was limited despite differences in porosity caused by variations in material throughput. However, in the evaluation of environmental conditions, pore defects were concentrated in the welds produced using filler wire whose surface moisture was not removed, resulting in a sharp decrease in tensile strength. This indicates that the deterioration of the mechanical performance of HDPE extrusion welds can vary depending not only on the numerical porosity value, but also on the size, distribution, concentration, and of pore formation. In particular, residual moisture on the surface of the filler wire can induce internal defects in the weld and have a critical effect on strength reduction. Therefore, in the extrusion welding process, not only the storage condition of the filler wire but also surface moisture removal and drying management before welding are essential.

### 3.3. Effect of Defect Volume Fraction on Tensile Strength of HDPE Base Material

To quantitatively evaluate the variation in tensile strength according to artificial defects, volume-controlled defects were machined at the center of the gauge length of HDPE base material tensile specimens. In the previous evaluation of extrusion welds, increased internal porosity and decreased tensile strength were observed with changes in material throughput. However, these results may include effects of welding process variables, such as molten state, pressing condition, weld geometry, and interfacial bonding. Therefore, in this experiment, the defect volume fraction was independently controlled under base material conditions, excluding welding process effects, to separately evaluate the effect of internal defect volume on tensile strength reduction.

The artificial defects were designed to have the same volume as simplified spherical defects representing actual pores. The defect volume was converted based on the volume of the gauge section of the tensile specimen and defined as the defect volume fraction. As shown in Figure 14, tensile strength decreased as the defect volume fraction increased. Under the defect-free base material condition, the average tensile strength was the highest, at approximately 25.12 MPa, whereas it decreased to approximately 24.34 MPa under the condition with the highest defect volume fraction. This corresponds to a reduction of approximately 3.11%.

These results show that an increase in internal defect volume alone can reduce the strength of HDPE tensile specimens, regardless of welding process variables. The defects are considered to reduce the effective load-bearing cross-sectional area and act as local stress concentration sites, thereby causing a decrease in tensile strength. In particular, the decreasing trend in tensile strength with increasing defect volume fraction was similar to the trend of increased porosity and reduced strength observed in actual extrusion-welded specimens. Therefore, the tensile strength reduction of extrusion welds can be interpreted as the combined result of indirect effects from welding conditions and direct effects from internal pores formed during welding.

However, the artificial defects used in this experiment were simplified defects with controlled volumes and do not fully reproduce the irregular pore shapes, multiple pore distributions, interfacial defects, and microstructural changes observed in actual welds. Equivalent volume therefore does not imply equivalent mechanical behavior. Nevertheless, these results quantitatively demonstrate the relationship between artificial defect volume fraction and tensile strength reduction for the prescribed defect geometry in HDPE base material, without the additional influence of welding process variables.

## 4. Conclusions

In this study, pore formation mechanisms in HDPE extrusion welds were investigated, and the effects of internal pores on mechanical properties were evaluated. Material throughput, filler wire storage condition, and artificial defect volume fraction were considered to distinguish the effects of process-induced pores, moisture-induced pores, and internal defect volume. The main conclusions are as follows.

First, internal pore formation increased with material throughput. The average porosity increased from 0.06% at 0.96 kg/h to 0.37% at 2.00 kg/h and then slightly decreased to 0.33% at 2.60 kg/h, indicating a nonlinear relationship between material throughput and porosity. Increased material throughput is considered to reduce the residence time in the barrel, thereby limiting gas release from the molten filler material.

Second, tensile strength was only slightly affected by porosity under different material throughput conditions. Although porosity increased by approximately 492% between the 0.96 kg/h and 2.00 kg/h conditions, the tensile strength difference remained within approximately 1.49%. In contrast, elongation decreased more clearly with increasing porosity, indicating that internal pores more strongly affect ductility and deformation capacity than ultimate tensile strength within the investigated porosity range.

Third, residual surface moisture on the filler wire had a critical effect on weld quality. The filler wire stored in water and used after surface moisture removal showed weld bead morphology and tensile strength similar to those of the normally stored wire. However, when surface moisture was not removed, severe internal pores were formed, and tensile strength decreased by approximately 55.53%. This confirms that residual surface moisture during welding, rather than moisture absorption into HDPE, is the main cause of strength reduction.

Fourth, the artificial defect experiment showed that internal defect volume can directly reduce tensile strength. As the artificial defect volume fraction increased, the tensile strength of the HDPE base material decreased from approximately 25.12 MPa to 24.34 MPa, corresponding to a reduction of about 3.11%. This supports that internal pores decrease the effective load-bearing area and act as stress concentration sites.

Overall, pore formation in HDPE extrusion welding is affected by both process conditions and environmental factors. Therefore, material throughput should be controlled to ensure sufficient residence time for gas release, and surface moisture on the filler wire should be removed before welding. These findings provide practical guidance for reducing internal pores and improving quality control in HDPE extrusion welding.

## Figures and Tables

**Figure 1:**
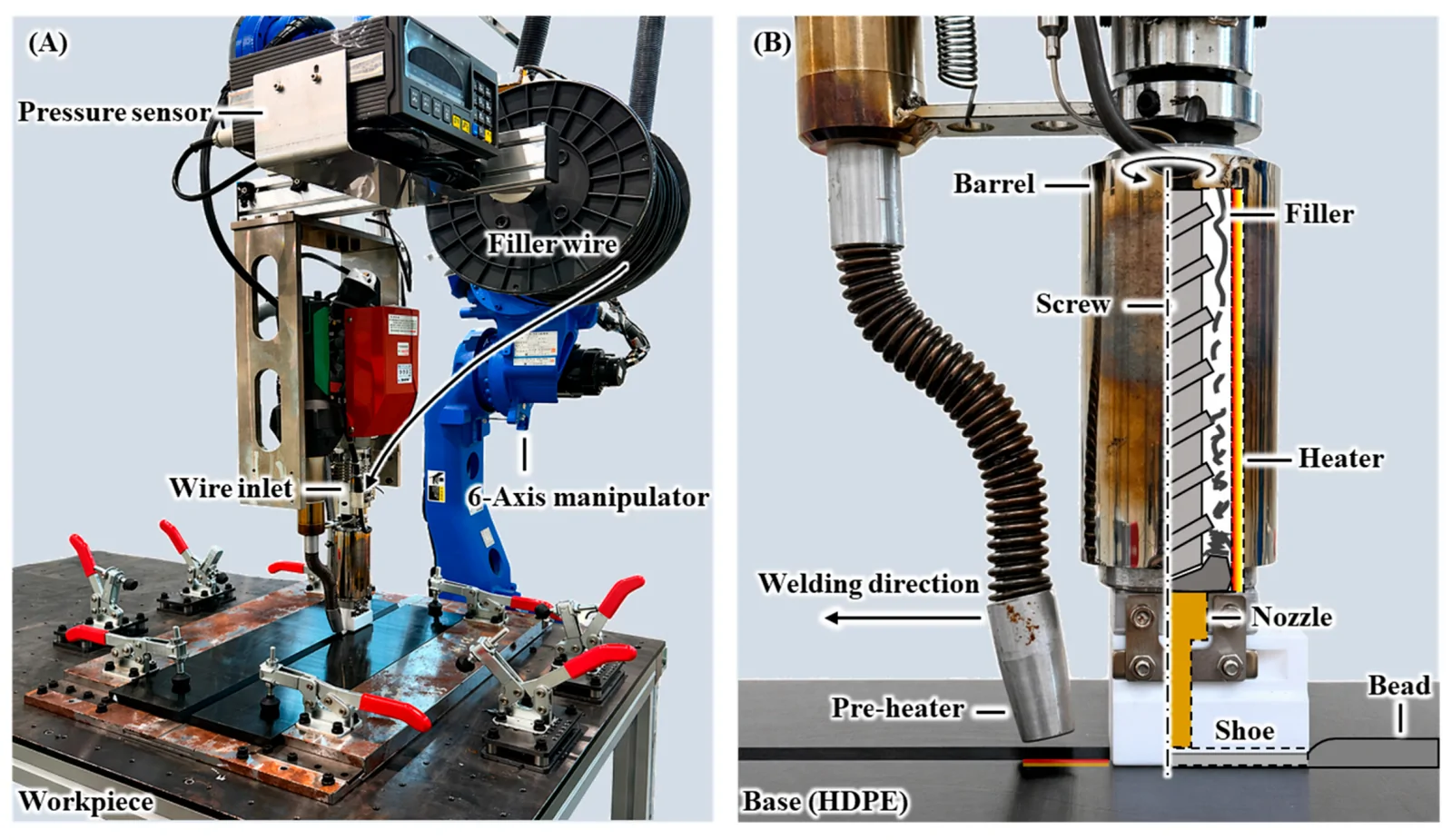
Automated extrusion welding system: (**A**) experimental setup; (**B**) schematic of the HDPE extrusion welding process.

**Figure 2:**
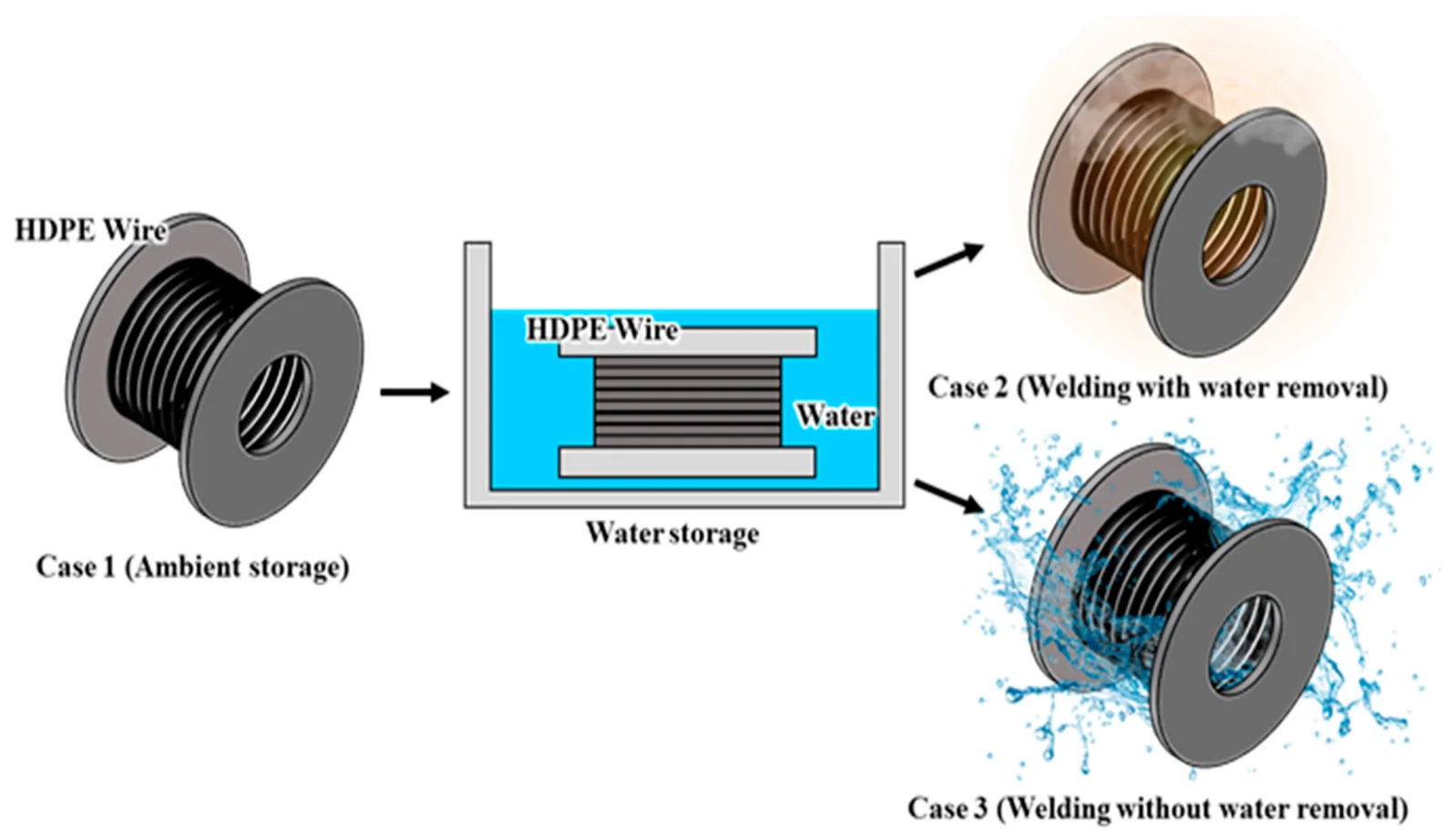
Schematic representations of specimens.

**Figure 3:**
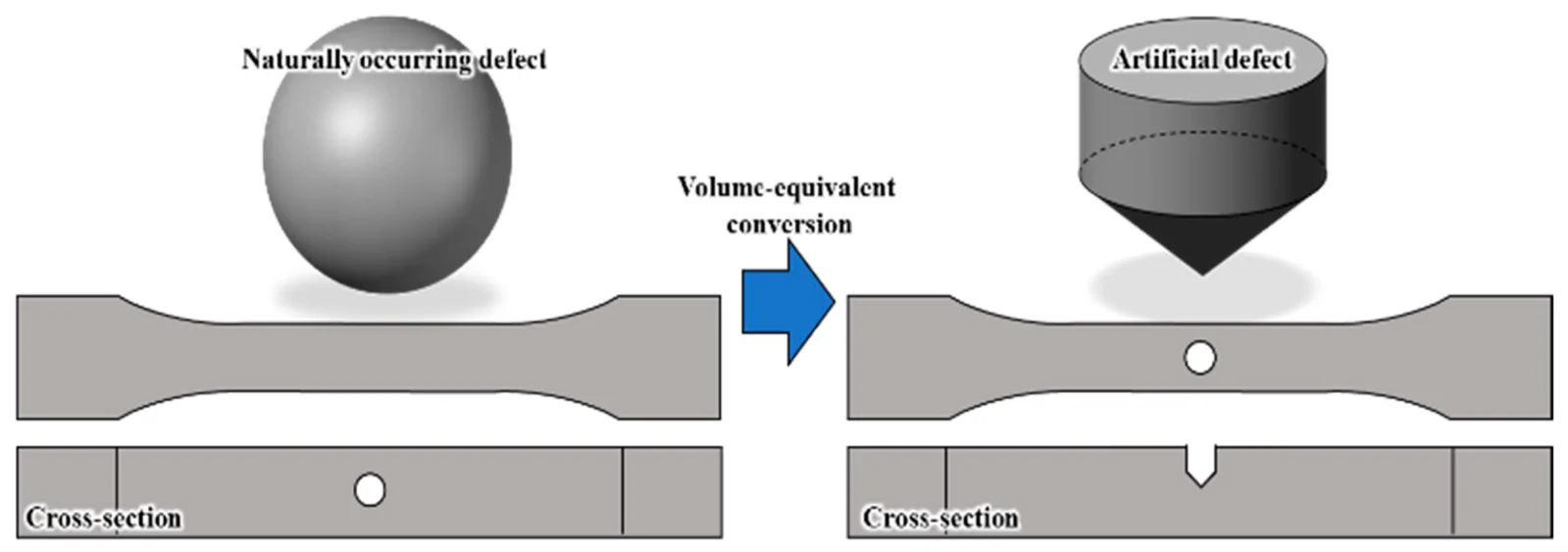
Schematic of volume-equivalent artificial defect design.

**Figure 4:**
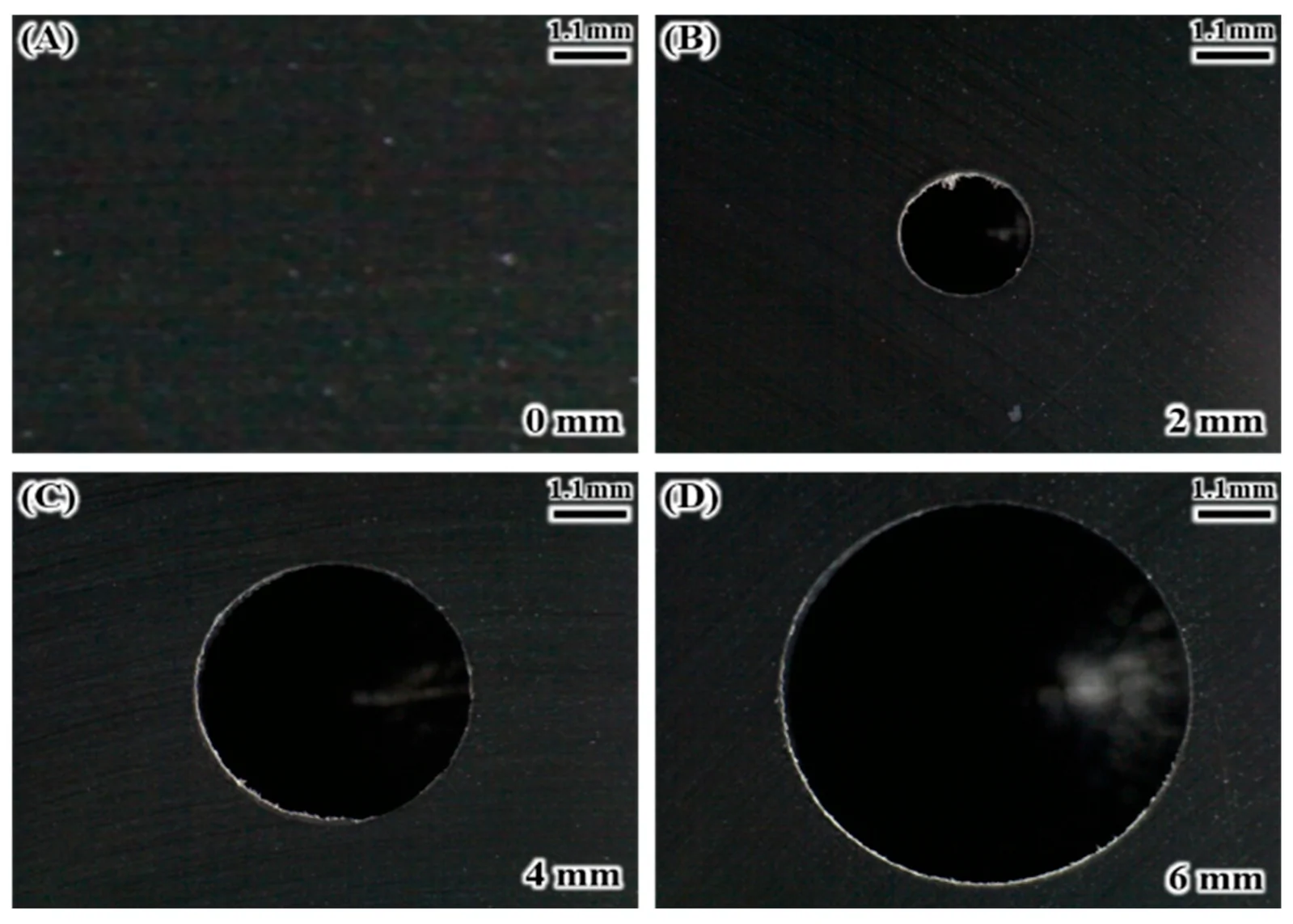
Fabricated artificial defects with varying diameters; (**A**) base material, (**B**) 2 mm, (**C**) 4 mm, and (**D**) 6 mm.

**Figure 5:**
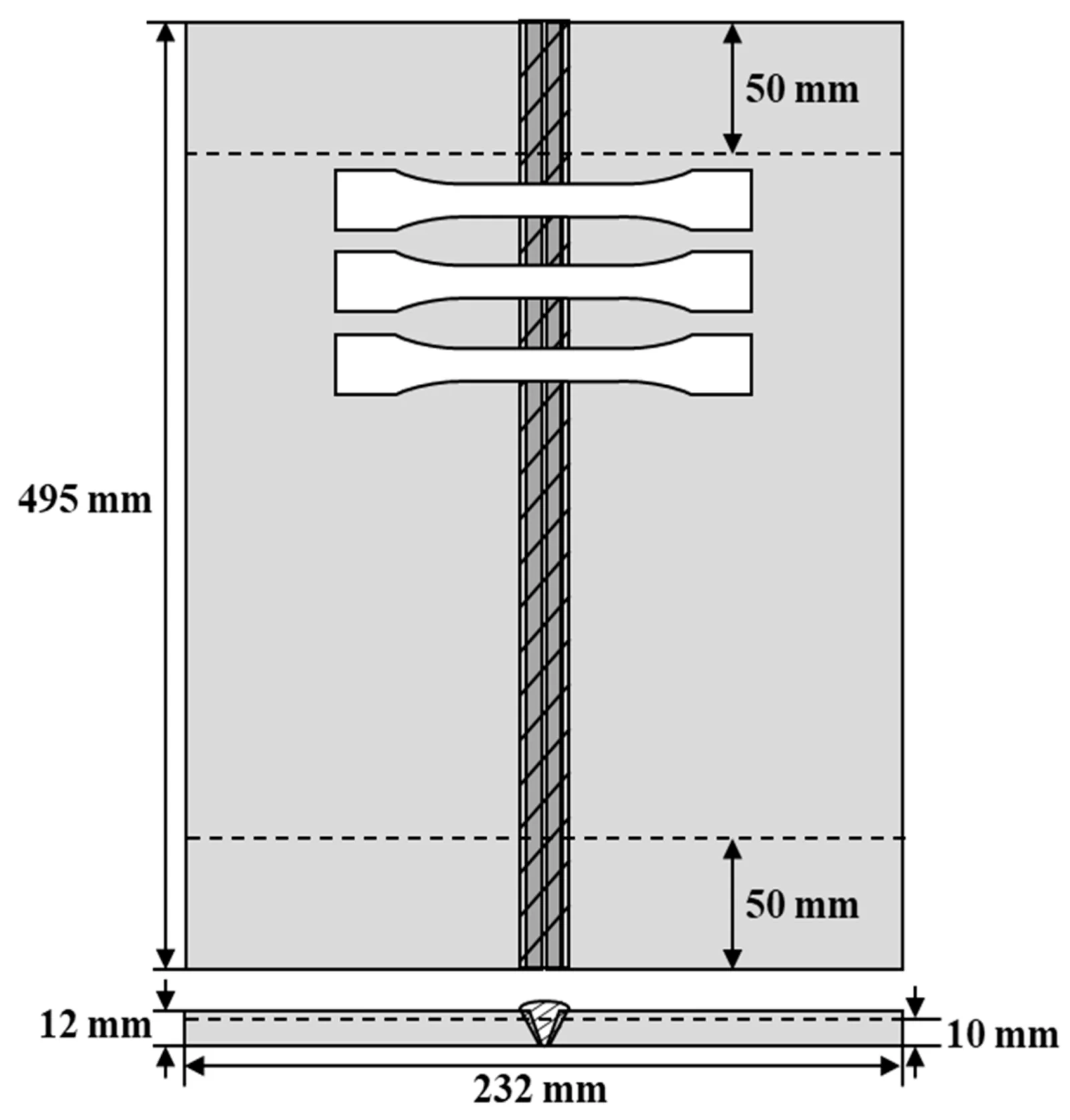
Preparation of tensile specimens.

**Figure 6:**
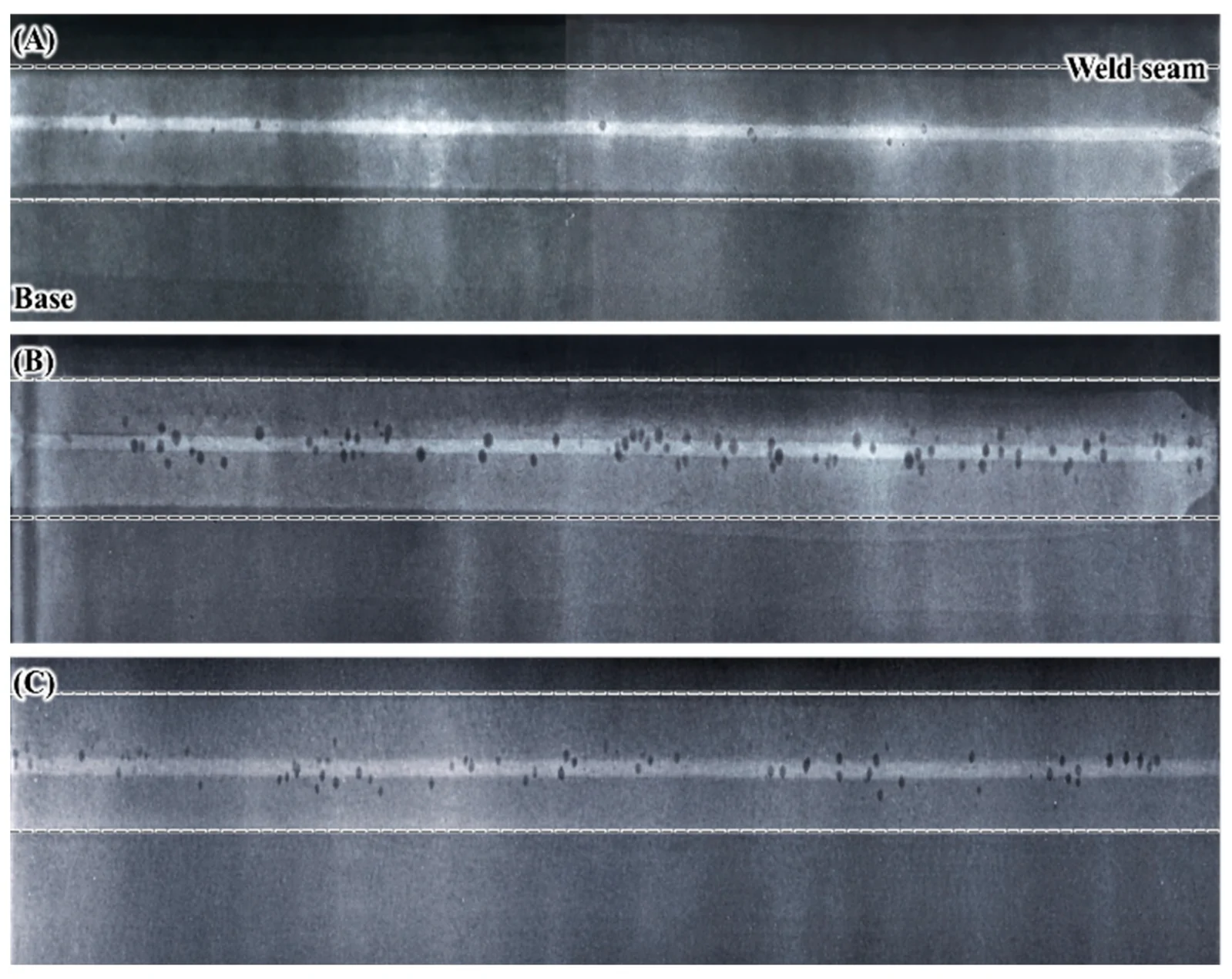
RT images of HDPE welds under different material throughput conditions; (**A**) 0.96 kg/h, (**B**) 2.00 kg/h, and (**C**) 2.60 kg/h.

**Figure 7:**
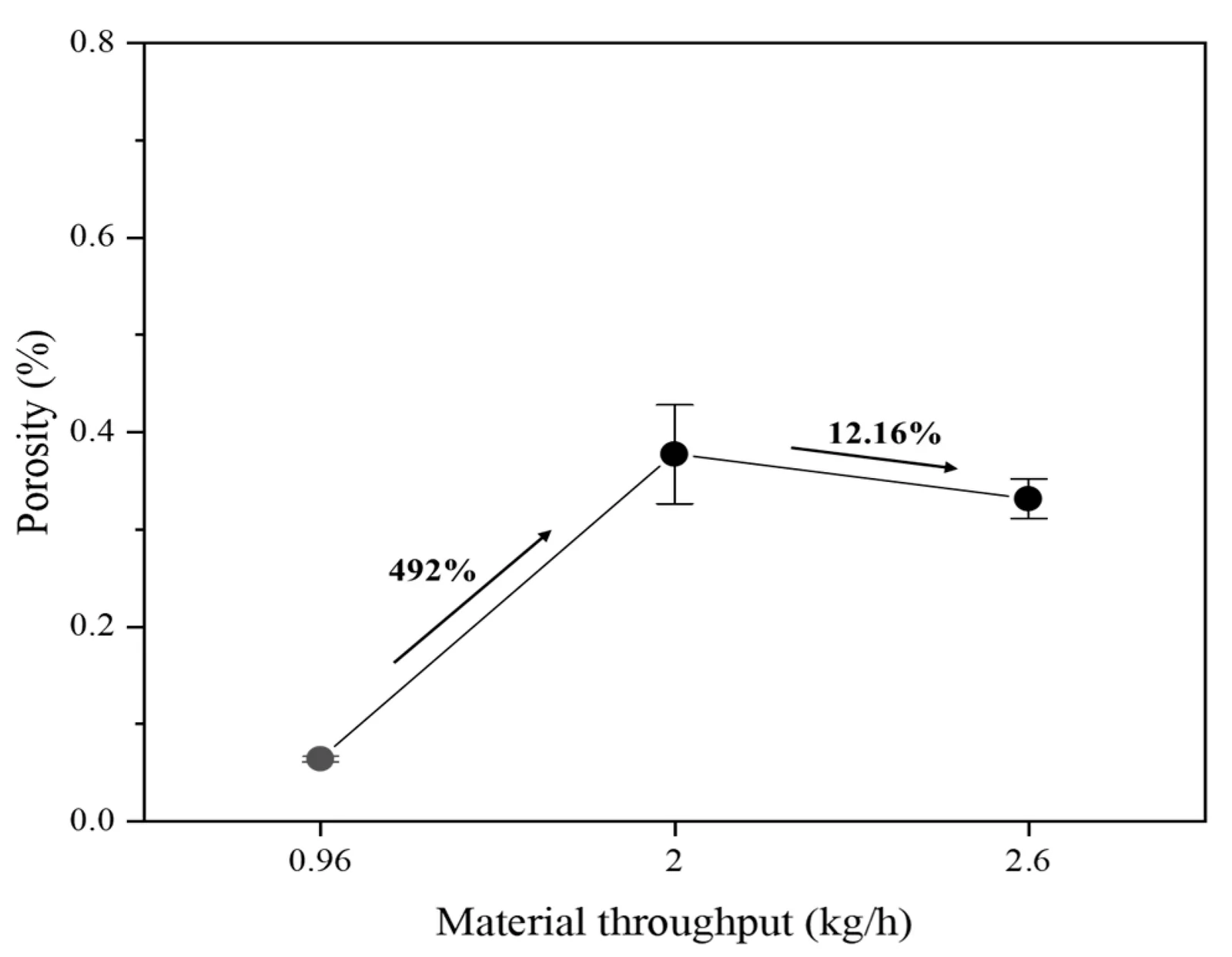
RT Effect of material throughput on porosity.

**Figure 8:**
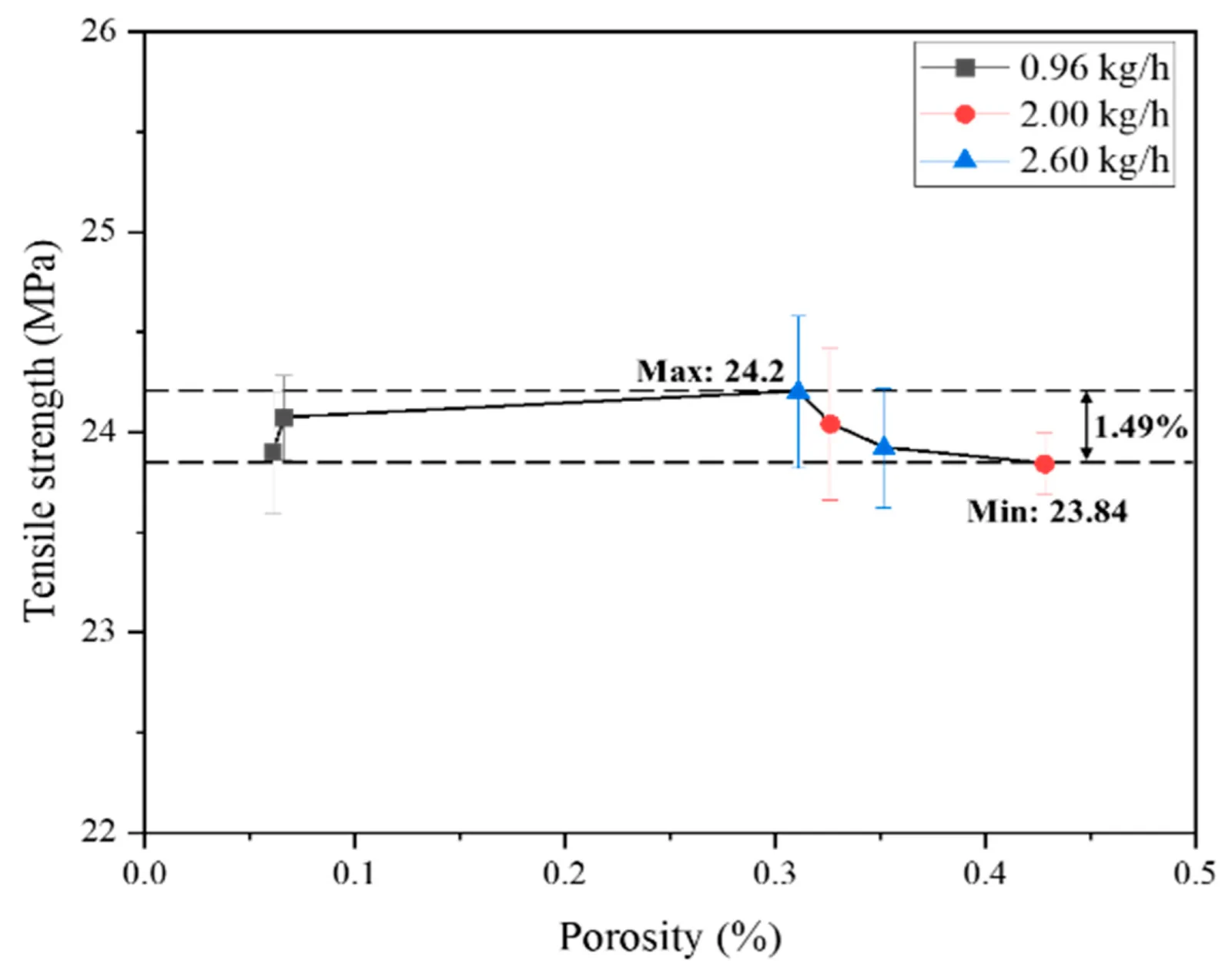
Effect of porosity on tensile strength.

**Figure 9:**
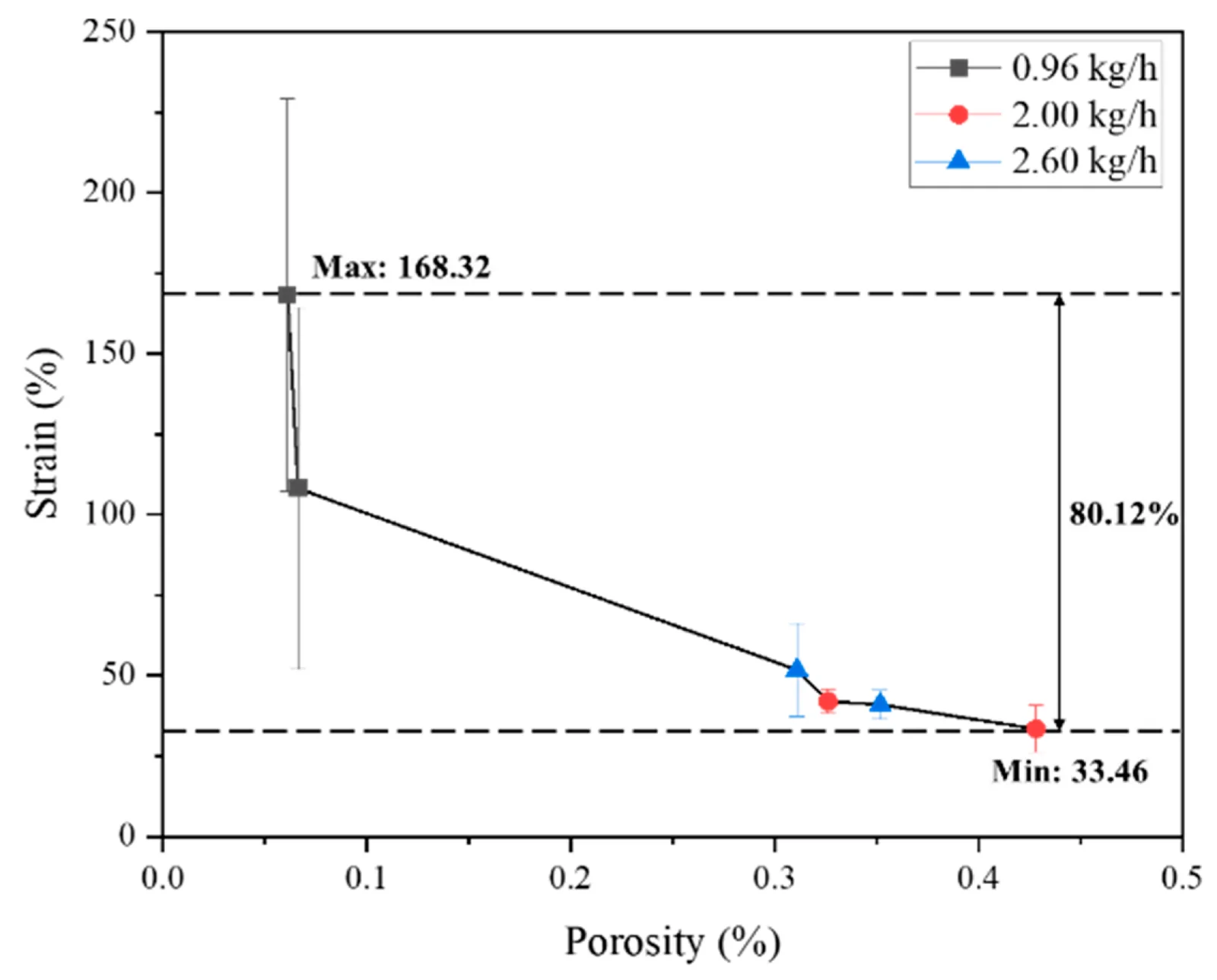
Effect of porosity on strain.

**Figure 10:**
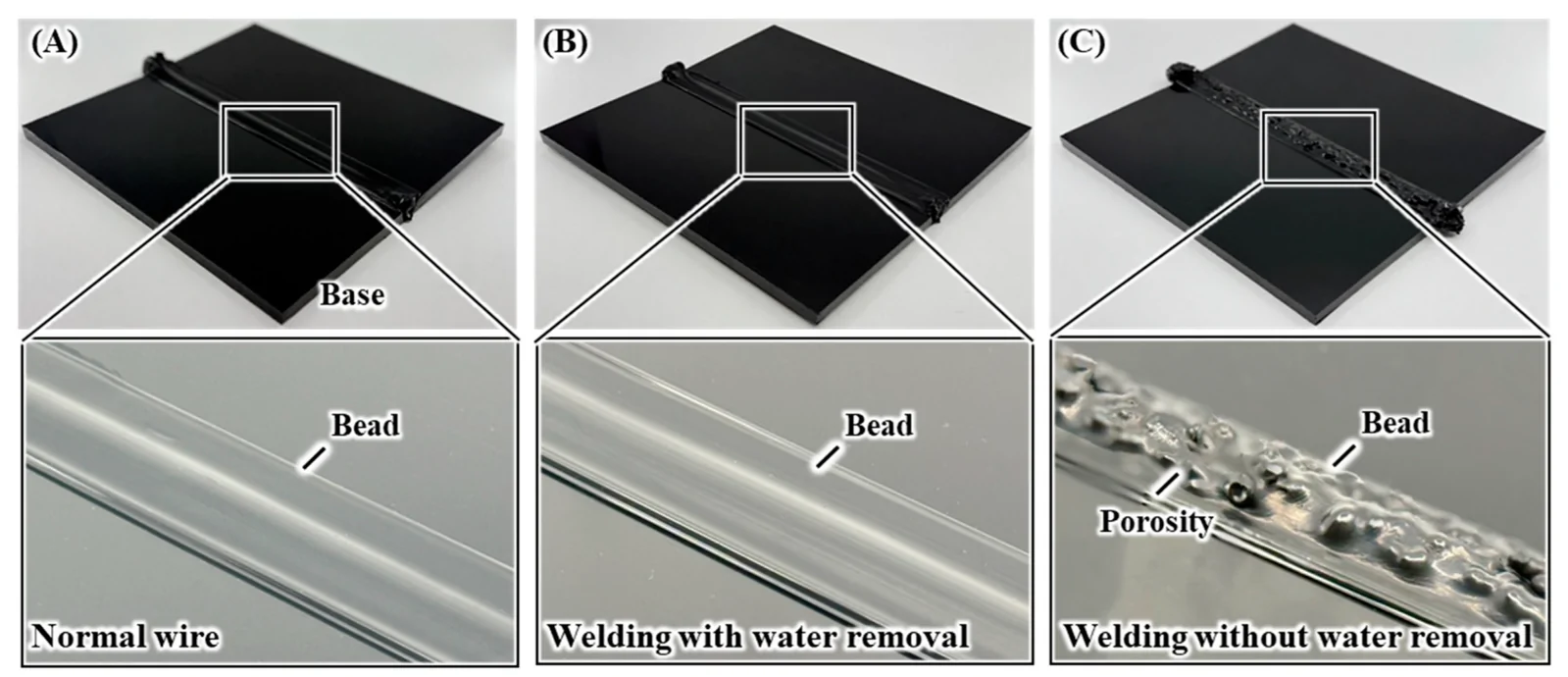
Weld bead surface observations under different environmental conditions; (**A**) normal wire, (**B**) welding with water removal, and (**C**) welding without water removal.

**Figure 11:**
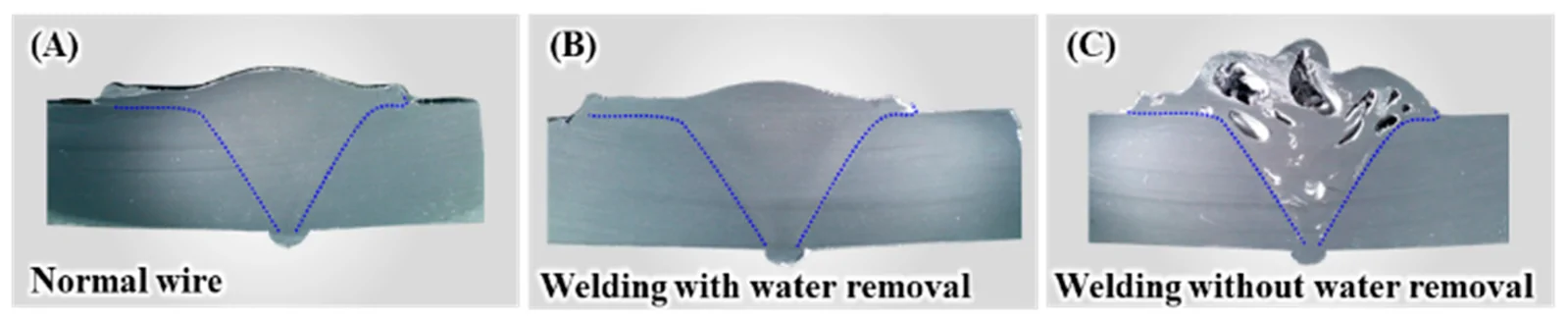
Cross-sectional morphology of weld beads under different storage conditions; (**A**) normal wire, (**B**) welding with water removal, and (**C**) welding without water removal.

**Figure 12:**
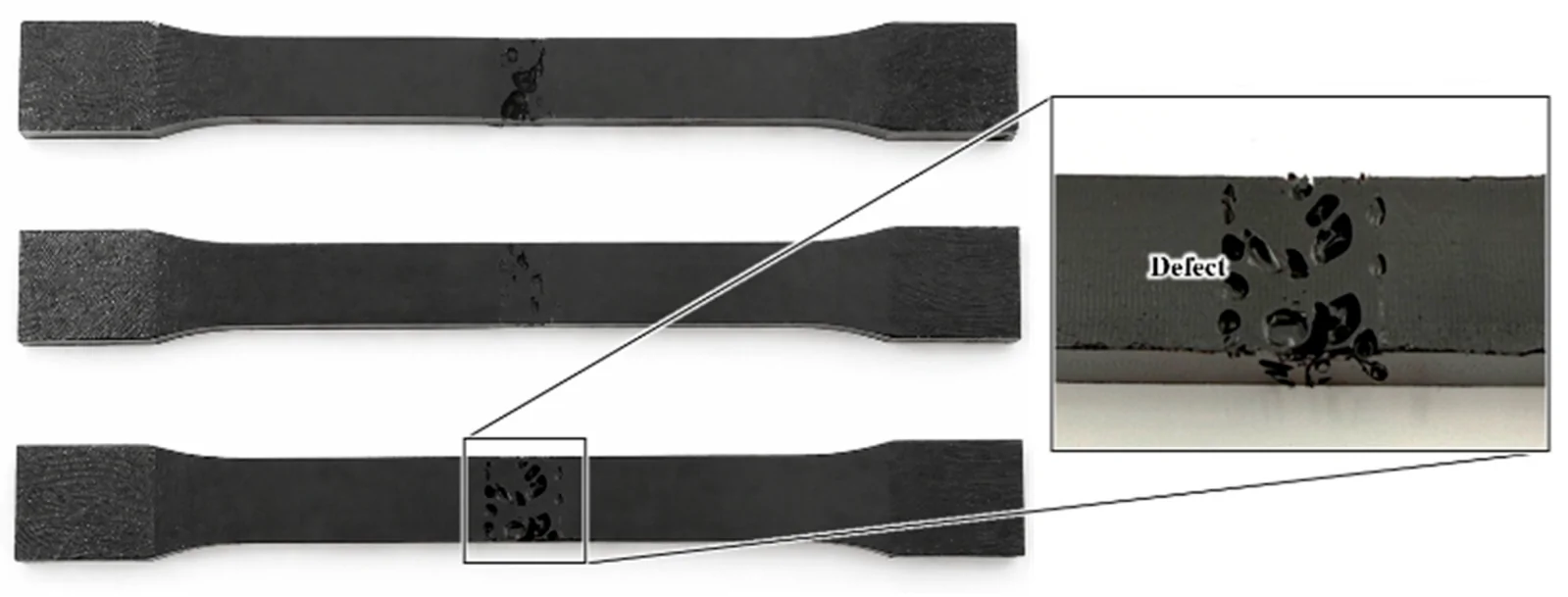
Tensile specimens fabricated under the welding without water removal condition and surface defects observed on the specimens.

**Figure 13:**
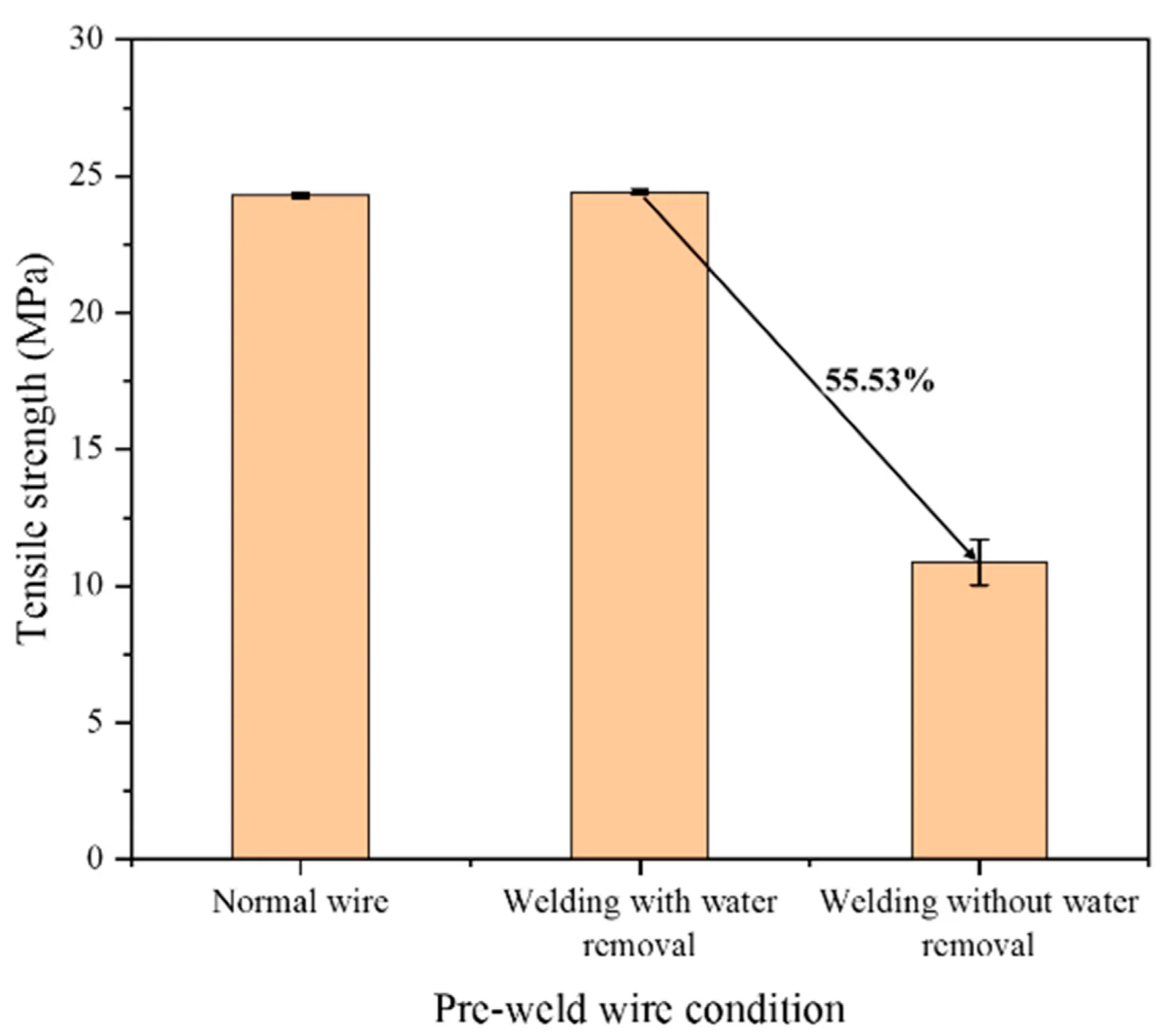
Tensile strength results of HDPE extrusion welds under different filler wire storage conditions.

**Figure 14:**
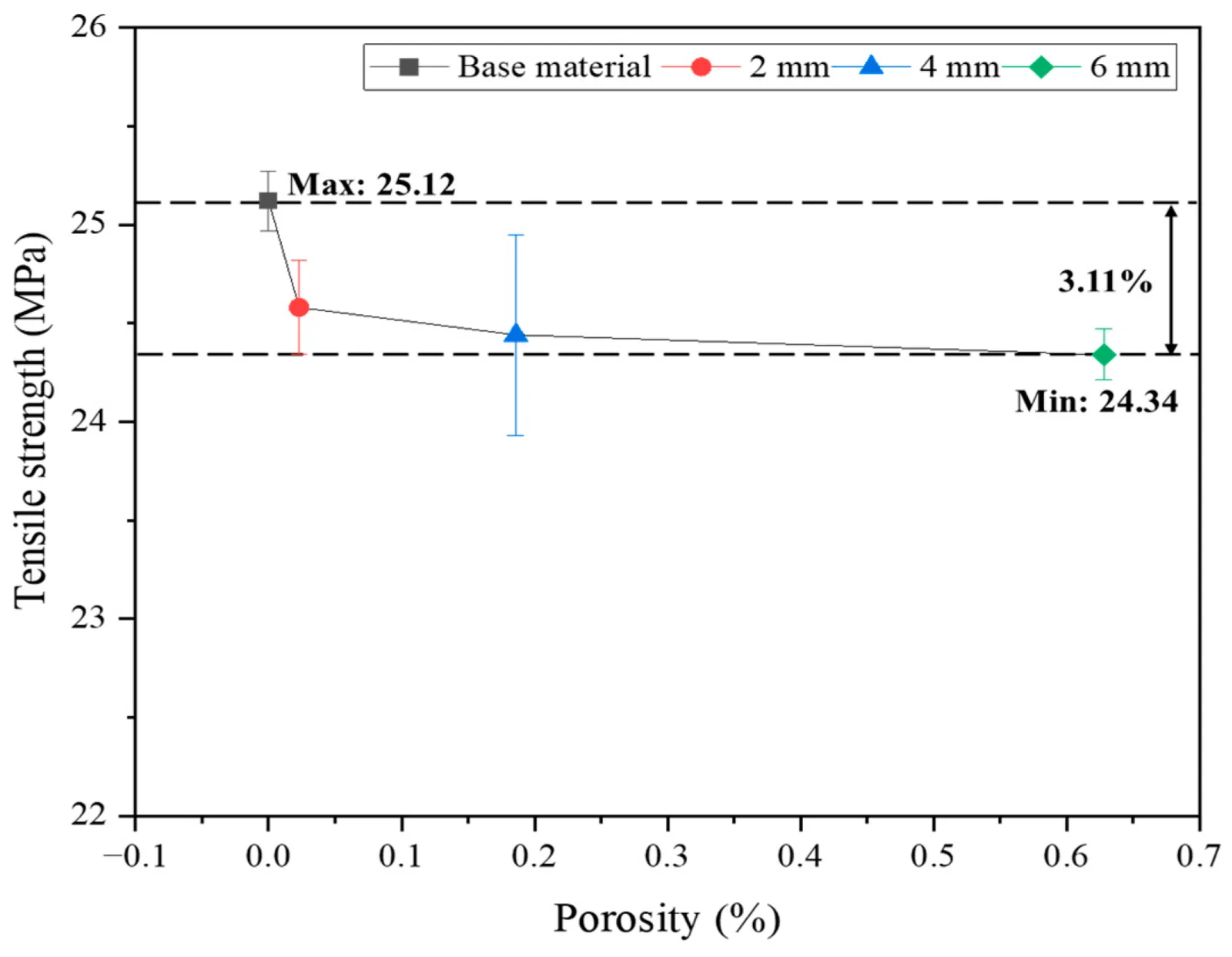
Effect of artificial defect volume fraction on the tensile strength of HDPE base material.

**Table 1:** Welding conditions under different material throughput conditions.

No.	Case 1	Case 2	Case 3
Material throughput (kg/h)	0.96	2.00	2.60
Welding speed (cm/min)	15	25	35
Melting temperature (°C)	240		
Preheat temperature (°C)	5~20		
Welding pressure (kgf)	250		

**Table 2:** Welding conditions under different filler material storage conditions.

No.	Welding Wire Storage Conditions		
Case 1	Room	10 °C	72 h
Case 2	Water	10 °C	72 h (Welding with water removal)
Case 3	Water	10 °C	72 h (Welding without water removal)
Material throughput (kg/h)			0.96
Welding speed (cm/min)			15
Melting temperature (°C)			240
Preheat temperature (°C)			5~20
Welding pressure (kgf)			250

**Table 3:** Material properties of the HDPE base material and filler material.

Type	Material	Melt Index (190 °C/5 kg)	T_m_ (°C)	T_c_ (°C)
Base material	HDPE	0.22	130	120
Filler material	HDPE	0.23	130	120

**Table 4:** Results of bead size measurements under different storage conditions.

No.	Storage Condition	Width (mm)	Height (mm)
Case 1	normal wire	24.75	3.2
Case 2	welding with water removal	25.03	3.05
Case 3	welding without water removal	25.98	6.88

## Data Availability

The data presented in this study are available upon reasonable request from the corresponding author.

## Abbreviations

The following abbreviations are used in this manuscript:

HDPE	High-density polyethylene
FRP	Fiber-reinforced plastic
PE	Polyethylene
PP	Polypropylene
PVC	Polyvinyl chloride
RT	Radiographic testing
CNC	Computer numerical control
DVS	German Welding Society
EN	European Standard
